# Identifying Key Topics Bearing Negative Sentiment on Twitter: Insights Concerning the 2015-2016 Zika Epidemic

**DOI:** 10.2196/11036

**Published:** 2019-06-04

**Authors:** Ravali Mamidi, Michele Miller, Tanvi Banerjee, William Romine, Amit Sheth

**Affiliations:** 1 Computer Science and Engineering Wright State University Dayton, OH United States; 2 Department of Biological Sciences Wright State University Dayton, OH United States; 3 Kno.e.sis Computer Science and Engineering Wright State University Dayton, OH United States

**Keywords:** social media, machine learning, natural language processing, epidemiology, Zika, infodemiology, infoveillance, twitter, sentiment analysis

## Abstract

**Background:**

To understand the public sentiment regarding the Zika virus, social media can be leveraged to understand how positive, negative, and neutral sentiments are expressed in society. Specifically, understanding the characteristics of negative sentiment could help inform federal disease control agencies’ efforts to disseminate relevant information to the public about Zika-related issues.

**Objective:**

The purpose of this study was to analyze the public sentiment concerning Zika using posts on Twitter and determine the qualitative characteristics of positive, negative, and neutral sentiments expressed.

**Methods:**

Machine learning techniques and algorithms were used to analyze the sentiment of tweets concerning Zika. A supervised machine learning classifier was built to classify tweets into 3 sentiment categories: positive, neutral, and negative. Tweets in each category were then examined using a topic-modeling approach to determine the main topics for each category, with focus on the negative category.

**Results:**

A total of 5303 tweets were manually annotated and used to train multiple classifiers. These performed moderately well (F1 score=0.48-0.68) with text-based feature extraction. All 48,734 tweets were then categorized into the sentiment categories. Overall, 10 topics for each sentiment category were identified using topic modeling, with a focus on the negative sentiment category.

**Conclusions:**

Our study demonstrates how sentiment expressed within discussions of epidemics on Twitter can be discovered. This allows public health officials to understand public sentiment regarding an epidemic and enables them to address specific elements of negative sentiment in real time. Our negative sentiment classifier was able to identify tweets concerning Zika with 3 broad themes: *neural defects*,*Zika abnormalities*, and *reports and findings*. These broad themes were based on domain expertise and from topics discussed in journals such as *Morbidity and Mortality Weekly Report* and *Vaccine*. As the majority of topics in the negative sentiment category concerned symptoms, officials should focus on spreading information about prevention and treatment research.

## Introduction

### Background

Zika was discovered in 1947 in Uganda [1]. From the 1960s to 1980s, only 14 cases were diagnosed across Asia and Africa, and it typically caused mild symptoms [2]. The first large outbreak occurred in 2007, with the virus spreading from Yap across the Pacific with cases reporting mild symptoms. However, cases were likely underreported from 1947 to 2008 because the symptoms were similar to chikungunya and dengue. It was not until this most recent outbreak that Zika was linked to Guillain-Barré syndrome and microcephaly [1]. Owing to the new-found association of Zika and neurological disorders, people started expressing concern with the Zika virus, especially after an article in the British Broadcasting Corporation (BBC) stated that the United States declared the Zika virus scarier than first thought [3].

In our previous exploratory study [4], we collected 1.2 million tweets over a period of 2 months and developed a 2-stage classifier to categorize relevant tweets as concerning 4 disease categories: symptoms, treatment, transmission, and prevention. Tweets in each disease category were then examined using topic modeling to ascertain the top 5 themes for each category. We demonstrated how discussions on Twitter can be discovered to aid public health officials’ understanding of societal concerns. Our previous work focused on identifying relevant tweets with little emphasis on public sentiment. Much of the fear around Zika concerns the symptoms it causes [3]. Therefore, in this study, we turn our focus toward an in-depth analysis of the symptoms of Zika and undertake an analysis of specific positive, negative, and neutral sentiments expressed about the Zika virus.

### Related Works

Identifying sentiment on a specific topic was pioneered by Chen et al [5,6]. Since then, several studies have looked at sentiment analysis on a variety of topics. Overall, 2 studies focused on personal communication tweets only [7,8]. The study by Daniulaityte et al [7] collected 15,623,869 tweets from May to November 2015 using keywords related to synthetic cannabinoids, marijuana concentrates, marijuana edibles, and cannabis. They found that using personal communication tweets only, compared with all tweets, improved binary sentiment classification (negative and positive) but not multiclass classification (positive, negative, and neutral). A study by Ji et al [8] collected tweets concerning listeria from September 26 to 28 and October 9 to 10 in 2011. They also focused on personal communication tweets only for sentiment classification (negative and not negative) and also found that the classifiers performed well after excluding nonpersonal communication (with a classification of F1 score=0.82-0.88). Instead of focusing on personal communication tweets alone, we included all relevant tweets after the BBC article about scientists declaring Zika scarier than initially thought [3] in our previous study [4]. A study by Househ collected approximately 26 million tweets and Google News Trends concerning the Ebola virus from September 30 to October 29, 2014 [9]. This study also influenced the decision to use all tweets and not just personal communication when they found that news feeds were the largest Twitter influencers during the Ebola outbreak.

Ghenai and Mejova [10] collected 13,728,215 tweets concerning Zika from January to August 2016. Tweets were annotated as debunking a rumor, supporting a rumor, or neither. They concluded that mainstream news websites may help spread misinformation and fear. A study by Seltzer et al [11] collected 500 images from Instagram from May to August 2016 using the keyword *Zika*. Of those 500 images, only 342 were related to Zika. Of those 342 images, 193 were coded as *health* and 299 were coded as *public interest*. Of the *health* images, the majority related to transmission and prevention, which is similar to what we found in our previous study on Twitter [4]. This shows results can be corroborated across different social media platforms. Seltzer et al also found that many of the images portrayed negative sentiment and fear. Their study was limited to using images and was only concerned with negative sentiment. Our study will use tweets and will include positive, neutral, and negative sentiment.

In many of these studies, the main topical content within each sentiment category was not explored. We take this additional step in our study to determine the topics of public concern regarding the Zika virus. We also used all tweets including personal communication as well as news articles because news articles can go viral and include negative sentiment, as seen with the BBC article briefly described in the background section [3]. The phenomenon of news articles going viral and including negative sentiment is also discussed in our previous study [4].

### Purpose of the Research

**Figure 1:**
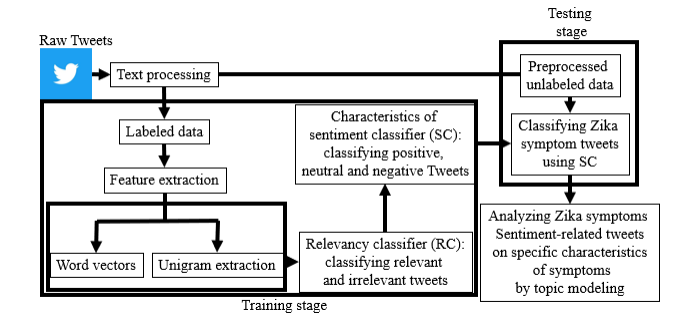
Block diagram of content retrieval using two-stage supervised classification followed by unsupervised analysis for characteristics of sentiment content.

In this study, public sentiment concerning the Zika virus symptoms was explored to determine important topical subcategories for positive, neutral, and negative tweets. Using the framework shown in Figure 1, 2 main research questions (RQs) were addressed:

RQ1a: Data Annotation Analysis: What was the distribution of positive, neutral, and negative tweets in the gold standard dataset? What was the agreement between the 2 annotators’ labels used as the gold standard for the sentiment classification?

RQ1b: Classification Performance: How well can we categorize tweets as positive, neutral, and negative in an automated fashion?

RQ2: Topical Analysis: What were the main topics discussed in the 3 sentiment categories with a focus on the negative sentiment category?

**Figure 2:**
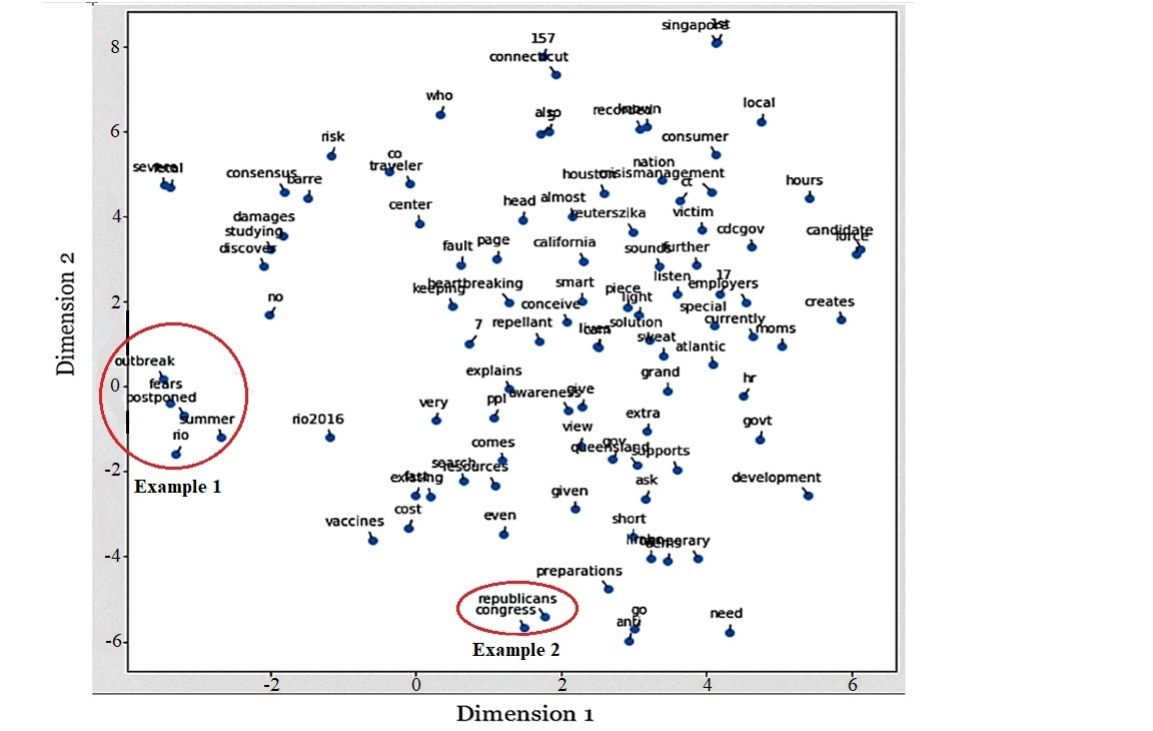
Visualization of Zika word embedding using t-SNE which shows clusters of related word groups within the context of Zika tweets.

## Methods

### Data Collection

This study utilizes data obtained in a previous study [4] using Twitris 2.0, a semantic Web application that aids comprehension of social perceptions by semantics-based processing of massive volumes of event-centric data on social media [12]. In the previous study, 1.2 million tweets were collected between February 24, 2016, and April 27, 2016, using the keywords *Zika*, *Zika virus*, and *Zika virus treatment* [4]. Before analysis, tweets were preprocessed by removing non-American Standard Code for Information Interchange (ASCII) characters, capital letters, retweet indicators, numbers, screen handles (@username), punctuation, URLs, whitespaces, single characters such as *p* that do not convey any meaning about topics in the corpus, and stop words such as *and*, *so*, etc. A random sample of 1467 tweets was annotated as relevant versus not by 3 microbiology and immunology experts and used as the relevancy ground truth. All tweets were then classified as relevant or not using the relevancy ground truth and several supervised classification techniques along with bootstrapping (bagging) techniques. The performance of the classifiers was assessed using tenfold cross-validation with average precision, recall, F1 score, and area under the curve being reported. The multinomial Naive Bayes classifier performed best with an area under the curve of 0.94. Another random sample of 1135 relevant tweets was annotated by the same 3 microbiology and immunology experts to use as the disease characteristics (DC; symptoms, treatment, transmission, and prevention) ground truth. The relevant tweets were then classified into 1 of the 4 DC categories using the DC ground truth and the same supervised classification techniques and performance measures used for relevancy classification. The multinomial Naive Bayes classifier performed best again with areas under the curve ranging from 0.83 to 0.94. This resulted in 48,734 tweets being classified as symptoms, 9937 tweets as treatment, 101,539 tweets as transmission, and 101,456 tweets as prevention [4]. As the Zika symptoms were of public concern, this study focuses on determining the sentiment of those 48,734 tweets collected and classified as discussing Zika symptoms in our previous study.

We have built upon that model described in [4] to explore the sentiments associated with the symptoms category. In this study, we used n-grams–based logistic regression to classify tweets as positive, negative, or neutral. The top themes in each sentiment category were then determined using latent Dirichlet allocation. This allowed us to better explore the themes in each sentiment category so public health officials can address the topics of public concern, such as neurological defects.

To address the RQs, we built the following methodological framework in Figure 1. The 48,734 tweets were preprocessed and labeled as positive, negative, and neutral. Features were then extracted using word embeddings and n-grams. A 2-staged classifier was built using the extracted features to identify the relevant tweets and then categorize them into the 3 sentiment categories. Preprocessed unlabeled tweets in each sentiment category were then analyzed using topic modeling techniques to find the top 10 topics for each of the 3 sentiment categories. This process is useful for discovering public sentiment regarding disease outbreaks and addressing apprehensions in real time.

### Data Annotation Analysis (Addressing RQ1a)

A total of 5303 random tweets selected from a total of 48,734 tweets were annotated as positive, neutral, or negative by 2 annotators with domain knowledge related to Zika epidemics. A tweet was considered positive if it mentioned research discoveries related to Zika, as seen in this tweet: “#Zika structure discovered, raising hopes for new ways to combat virus” or reflected a positive attitude toward treatments, preventions, or funding for Zika as seen in this tweet “#Bayer scientists aiding in fight against #Zika virus.” A tweet was considered negative if it discussed the defects/disorders caused by Zika such as “CDC confirms Zika virus causes severe birth defects #business”, discusses the spread of Zika as seen in this tweet “#news Zika virus may spread to Europe in coming months, WHO warns #til_now #Reuters.” Tweets were considered neutral if they gave information with no emotionally charged wording such as *hope*, *combat*, and *severe* or the overall sentiment of the tweet was neutral. Examples of neutral tweets are “Zika symptoms, diagnosis and treatment, from the CDC #ZikaVirus” and “WHO: #Zika situation report, March 31.” Agreement was found using the Cohen's kappa, which is a robust statistic useful for either interrater or intrarater reliability testing and accounts for the possibility of guessing [13]. These tweets became known as the gold standard dataset once significant agreement was reached (Kappa >.81) [13].

#### Preprocessing

Before data analysis could begin, tweets had to be preprocessed by removing screen handles (@username), URLs, non-ASCII characters, and retweet indicators. Tweets were then further processed by removing single letters such as *a*, *e*, and *i*; extra spaces; and stop words. Stop words are the most commonly used words in the English language such as *and*, *in*, and *for*. This preprocessed tweet corpus was used for extracting features using the word embeddings and n-grams. These features were extracted similarly to our earlier studies [4,14].

##### Word Embedding (Feature Extraction)

Machine learning algorithms are incapable of handling raw text or strings and require numeric data to extract knowledge from textual data and build applications. Word embedding is a technique that maps individual words to a predefined vector space in such a way that the semantic relation between words is preserved [15].

In addition, words or phrases from the tweets were embedded into the n-dimensional space where n is the number of words in the corpus. After word embedding, a sentence can be considered as a sequence of points that are grouped according to a semantic criterion so that 2 similar words are close to each other. It captures the context of words, while reducing the number of features in the data. To provide a better understanding of word embedding, we provide an example from a sample of our dataset. For visualizing the high-dimensional data, we used a technique called t-distributed stochastic neighbor embedding, which maps each data point to a lower dimensional space (of size 2) [16]. From Figure 2, we see the spatial distribution of a random sample of 100-word embeddings generated from the Word2vec model [17]. This figure is based on a subset of random tweets and is included purely to show how words used in the same context are close to each other in the vector space. We see that words that are similar eventually come spatially closer in the vector space. For example, words such as *outbreak*, *fears*, *postponed*, and *summer* (example 1) are spatially close because they are used in the same context in the case of the Rio Olympics and words such as *republicans* and *congress* (example 2) are spatially close together as they are used in the context of Zika funding. The word embedding algorithm was used to generate features to help classify tweets as positive, negative, or neutral.

### Models

We used 2 different main models for classification. One was Word2vec [18] and the other was an n-gram model [14].

#### n-Gram Model

In this model, features were extracted from tweets using the Stanford Natural Language Processing Part of Speech tagger [19] and n-grams [20], where an n-gram represents a sequence of words treated as a single entity or feature. Initially, features were identified from the tweets and the count for each feature was determined. Only the top 20 unigrams and bigrams were used for classification because the corpus was large, and we only wanted to capture the most frequently used text features. In total, there were 61 features. Examples include *AT_Mention*, *Zika*, *Discourse marker*, *microcephaly*, *fetal*, *Pronoun*, *health*, *birth* *defects*, *Zika infection*, *Hashtag*, and *brain damage*.

#### Word2vec Model

Word2vec comprises 2 different methods: continuous bag of words (CBOW) and skip-gram [21]. In the CBOW method, the goal is to predict a word given the surrounding words, that is, the words before and after it [21]. Skip-gram is the opposite: we want to predict surrounding words given a single word [21]. The skip-gram method with negative sampling works best with the medium- or large-sized datasets [15]. As our dataset was considered medium sized [15], we used the skip-gram model with a negative sampling rate of 10.

For the word embeddings, we used the Gensim library version 2.2.0 of Python version 3.5.4 [22] for converting all the words to an n-dimensional space before training the classifiers. The tokenized words were then fed to the Word2vec tool and trained with the skip-gram model. We considered a window size of 4 because the average length of the tweets was less than 10 words, which means 4 tokens apart from the target words are considered as adjacent words.

With these collective parameters, we generated the word vectors of size 300 and tested the learned vectors using the similarity functionality of the Word2vec. To evaluate the vectors generated using the tool, we selected 2 words *dengue* and *Zika*, which are mosquito-borne diseases, to assess similarity. Similarity is used to find the distance between 2 vectors. The closer the similarity is to 1, the more closely related the words are [23]. The similarity was 0.92, which indicates the words are closely related or used in a similar context. When words like microcephaly and pregnant were used, it gave related words such as woman, women, and infected, among others.

Vector operations such as sum and mean were used to build the final feature vector. The following are the operations performed on the word vectors:

Sum of Word Embeddings: This is the sum of all word vectors in the tweet. FV_Sum_=∑W

Mean of word Embeddings: Average of all the word vectors in the tweet. FV_Mean_=1/n ∑ W

*W* represents a single word in a tweet and FV_Sum_ and FV_Mean_ represent the feature vector of the tweets.

### Classification Performance (Addressing RQ1b)

Supervised classification algorithms, including logistic regression, support vector machines with radial basis function kernel, and random forest, were used for classifying the tweets into the 3 sentiment categories. These methods rely on labeled data, in this case, the 5303 randomly selected tweets that were annotated as positive, neutral, or negative by the 2 annotators from a total of 48,734 tweets. These classifiers were trained to categorize tweets into the specified categories based on the gold standard derived by the annotators.

The performance of each classifier was assessed using the stratified k-fold cross-validation as we had an unbalanced dataset. We report k=7 because there was no improvement in the result with increase of k and also it saves computation time. The stratified k-fold maintains equal number of samples for each annotator-labeled class [24]. In this method, 1 subsample (fold) of tweets was used for a testing set and the remaining 6 for training. This was repeated 7 times, with each subsample being used as the testing subsample once [24]. This study reports average recall (indication of category tweets not missed by the classifier), precision (correctly categorized tweets), and F1 scores (weighted average of precision and recall) as measures of classification performance for each classifier.

### Topical Analysis (Addressing RQ2)

Previous studies, such as the one by Lau, Collier, and Baldwin [25], have shown the usefulness of LDA for grouping text into themes in short text documents such as tweets. In this study, we used LDA topic modeling to identify the underlying topics discussed within each of the sentiment categories. In LDA, documents (tweets in this case) are represented as random mixtures over hidden topics, where each topic is characterized by a distribution over words that occur most frequently within that topic [26]. More specifically, LDA is a 3-level hierarchical Bayesian model, in which each word in a corpus is modeled as a finite mixture over an underlying set of topics. Each topic is then modeled as an infinite mixture over an underlying set of topic probabilities. The top words belonging to each topic are given as an output, and it is up to the researcher to interpret the topic’s meaning. This aids better qualitative exploration of the subtopics in each of the 3 categories.

To determine the number of topics required for topic modeling, we used perplexity, a measure used to evaluate topic models generated by LDA where the smaller the perplexity score, the better the generalization performance [22,26]. We used this measure to evaluate the topic modeling results by testing a range of 2 to 100 topic models for the 3 sentiment categories. For calculating the perplexity measure, preprocessed tweets were used. Words that occurred only once or twice in the corpus were removed as they increase the number of topics but will not give generalizable information [26].

## Results

In this section, the distribution of tweets in the gold standard dataset is discussed. The performance of 3 different classifiers using the Word2vec and n-gram models is also explained. Finally, the topic modeling results for the positive, neutral, and negative categories is explored with a focus on the themes that emerged in the negative sentiment category.

### Data Annotation Analysis (Addressing RQ1a)

**Figure 3:**
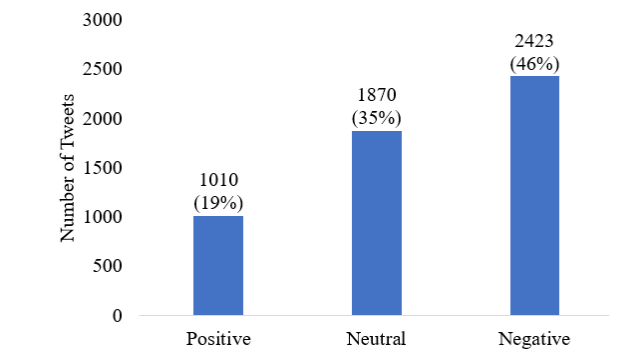
Distribution of tweets in three sentiment categories.

To train the classifiers, the gold standard dataset had to be created as described in the methods section above. The kappa value for the level of agreement between the 2 annotators was 0.95, indicating near-perfect agreement [13]. The distribution of the tweets in the gold standard dataset is shown in Figure 3. The majority of tweets displayed negative sentiment (2423; 46% of the total tweets) and the fewest displayed positive sentiment (1010; 19%). As can be seen in Figure 3, there is high class imbalance in the 3 sentiment categories.

### Classification Performance (Addressing RQ1b)

Table 1 provides the performance of the 2 text-processing models and the corresponding classifiers. The n-gram model performed slightly better than the word-embedding model. For this dataset, classifiers performed reasonably well, with F1 scores ranging from 0.48 to 0.68. However, the logistic regression classifier used with the n-gram model performed the best with an F1 score of 0.68. This performance is comparable with that in similar studies [7,18].

**Figure 4:**
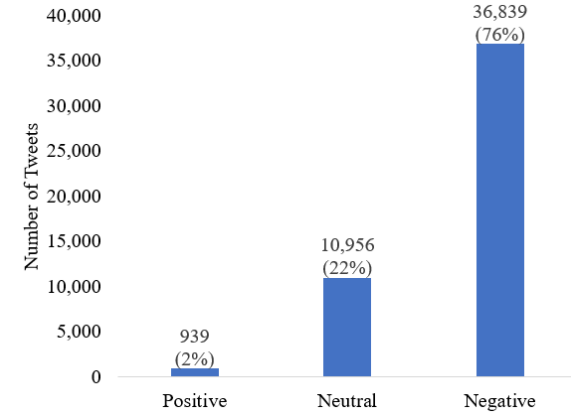
Number of tweets in three categories of the symptoms dataset (obtained from the n-gram based sentiment classifier).

Using the n-gram–based logistic regression sentiment classifier, we categorized all 48,734 tweets obtained from our previous study (Figure 4) [4]. The total number of negative tweets was almost 4 times larger than the positive and neutral categories combined. We can clearly see from Figure 4 that this is a highly unbalanced dataset, with the majority of tweets belonging to the negative sentiment category.

**Table 1:** Classifier performance for sentiment analysis using sevenfold cross-validation. The classifiers used are logistic, support vector machine, and random forest.

Classifier		Precision	Recall	F1 score
**Word2vec** **FV_Sum_ model**			
	Logistic regression	.68	.66	0.66
	Support vector machines	.67	.65	0.65
	Random forest	.55	.53	0.48
**Word2vec** **FV_Mean_ model**			
	Logistic regression	.63	.63	0.63
	Support vector machines	.66	.65	0.65
	Random forest	.50	.50	0.50
**n-gram model**			
	Logistic regression	.69	.68	0.68
	Support vector machines	.65	.65	0.65
	Random forest	.68	.67	0.67

### Topical Analysis (Addressing RQ2)

**Figure 5:**
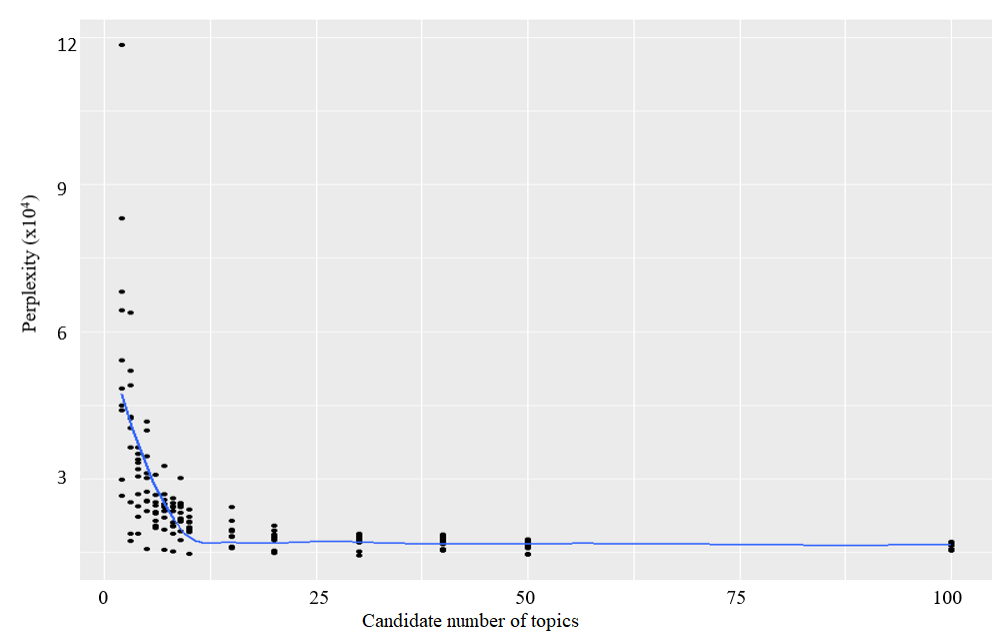
Perplexity plot measures for the 7-fold cross-validation of topic modeling for the negative sentiment category.

Within the tweets with negative sentiment, the perplexity decreased rapidly until about 10 topics and then leveled off (Figure 5). The perplexity graph for the positive and neutral category are available online [27]. This indicates that increasing the number of topics after 10 will not significantly improve the generalizability of the LDA models [26]. Therefore, 10 topics per sentiment were extracted.

The results of the LDA are discussed below for the positive, neutral, and negative categories. Themes and topics for all 3 sentiment categories were determined by an epidemiology expert based on the words given for each theme and some sample tweets containing those words. First, the topics for the positive and neutral categories will be briefly discussed. The tables, including the theme names, topic words, and example tweets for the positive and neutral topic models, are available online [27]. Then, a more detailed explanation of the negative sentiment topics will be presented.

### Topics From Positive and Neutral Sentiment

Within the positive sentiment themes, there were 4 broad qualitative topics within the 10 topics chosen using the perplexity measure with LDA: mosquito-killing methods, models to help understand the Zika virus, detection of the Zika virus in cells, and treatment and prevention discoveries (Table 2). These broader themes were labeled based on domain expertise and from journals such as Vaccine and MMWR, allowing further categorization of the 10 topics. For the broader theme of models that help understand the Zika virus, topic #1 contained tweets concerning a new model researchers were developing to study Zika pathogenesis and topic #2 described 3-dimensional (3D)–printed minibrains used for understanding the Zika virus. For the mosquito-killing methods theme, topic #4 contained tweets concerning sweat-emitting Brazilian billboards killing the Zika-carrying mosquitoes and topic #10 addressed other ways of killing Zika-carrying mosquitoes. In the treatment and prevention discoveries theme, topic #3 comprised tweets regarding the discovery of how Zika stunts the development of a fetus, topic #5 characterized the development of vaccines to treat Zika, and topic #8 reported about the IBM magic bullet to destroy all killer viruses. This *magic bullet* is actually a macromolecule that will attach to the surface of any virus and prevent it from attaching to a human cell [28]. If the virus cannot attach and enter a cell, infection is prevented. The macromolecule is also basic, neutralizing the acidity of an infected cell in case the virus is already infecting human cells by the time the *magic bullet* is used [28]. In the broader theme of detection of the Zika virus in cells, topic #6 regarded different types of tests for identifying Zika infection, topic #7 outlined the detection of Zika using fetal tissue, and topic #9 detailed the detection of Zika accumulations in the brain.

Table 2 Positive sentiment topic modeling results grouped together based on the broader themes. The numbers reflect the relative size of the theme. For example, the topic mouse model had more tweets than 3D-printed minibrains.

**Table 2:** Positive sentiment topic modeling results grouped together based on the broader themes. The numbers reflect the relative size of the theme. For example, the topic mouse model had more tweets than 3D-printed minibrains.

Topic		Words	Tweet
**Model broader theme**		
	#1 Mouse model	Researcher, mouse, model, develop, health, and research	new #zika *mouse model researchers develop* another *mouse >model* of zika infection that mimics the disease in humans
	#2 3-dimensional–printed Minibrains	Scientist, test, brain, mystery, and help	mini 3d printed *brains help scientists* understand zika virus
**Mosquito broader theme**		
	#4 Brazilian billboards	Rapid, billboard, emit, Brazilian, and structure	sweat- *emitting Brazilian billboards* lure zika-carrying mosquitoes to their death | mnn—mother nature network
	#10 Killing mosquitoes	Mosquito, infect, kill, insight, and biomolecular	researchers develop #algae to *kill* #*mosquitoes* carrying viruses like #zika
**Virus discovery broader theme**		
	#3 Fetal brain development	Fetus, human, discover, and help	how zika virus stunts *foetal* brain development researchers have *discovered* how hijacking a *human* immune mole…
	#5 Vaccines	Model, infect, vaccine, provide, and develop	mouse *models* of zika virus *infection* in pregnancy *provide* basis to *develop vaccines*, treatments
	#8 IBM magic bullet	Kill, develop, and understand	IBM research IBM announces magic bullet to zap all kinds of *killer* viruses, like #zika by seancaptain
**Detection broader theme**		
	#6 Zika tests	Urine, discover, pattern, Jamaica, programmable, and molecular	#salingfollow interim cdc guidance finds *urine* specimen better than serum for rapid and specific zika testing—cdc
	#7 Fetal tissue research	Fetal, tissue, infect, detect, equip, and test	last month, *fetal tissue* research helped doctors’ understand how the zika virus *infects fetus* & how to *detect* its presence much
	#9 Zika accumulation	Reveal, accumulate, Zika, virus, examine, pregnancy, and report	one of the first mouse models of #*zika reveals* the *virus accumulates* in the brain

Overall, the broader themes in Table 3 (model, mosquito, virus discovery, and detection) were present in the positive sentiment category because they all have to do with helping prevent transmission or research that could lead to treatments. Both of these topics reflect positive public perception because they help prevent the defects that have become associated with Zika. For example, tweets in the mosquito theme discussed ways to kill mosquitoes, which would help prevent the spread of Zika [29]. Tweets in the model and viral discovery themes addressed discoveries that could help lead to treatments, such as the IBM magic bullet [28]. Virus discovery tweets were positive because they pointed to faster ways to detect Zika. Knowing where Zika accumulates would help with developing treatments [30]. Tweets in the positive category also used words with positive connotations such as *understand*, *develop*, *hope*, *discover*, *benefit*, and *reveal*, among others. While themes in the positive sentiment category mainly addressed research to treat Zika and prevention methods, themes in the neutral category mostly comprised posts from news agencies stating facts.

Within the neutral sentiment topics, there were 3 broader qualitative themes: public health messages, knowledge gaps, and Zika characteristics (Table 3). In the public health messages, topic #1 explained how scientists were trying to unravel the Zika mystery, topic #2 cautioned about the dangers of Zika infection to pregnant mothers, topic #3 declared that Zika is a mosquito-borne disease, topic #4 specified the laws regarding birth control and abortion, topic #5 discussed fighting the mosquitoes, and topic #6 regarded the officials warning the public to be careful not to be bitten at work. Knowledge gaps consisted of topic #7, which discussed knowledge gaps concerning the Zika virus. In the Zika characteristics theme, topic #8 affirmed Zika symptoms, topic #9 included comparisons between dengue and Zika, and topic #10 described fetal brain damage from Zika infection.

**Table 3:** Neutral sentiment topic modeling results grouped together based on the broader themes. The numbers reflect the relative size of the theme.

Topic		Words	Tweet
**Public health messages broader theme**		
	#1 Zika mystery	Brazil, common, unravel, question, important, disease, and issue	#voanews *brazil* scientists seek to *unravel* mystery of zika twins scientists struggling to unravel t…
	#2 Aedes mosquito	Mosquito, infect, pregnancy, outbreak, women, and child	the zika virus and the dengue *mosquito* have a common nature. very resistant ones, and very dangerous too. *infects* mothers with *pregnancy*!
	#3 Mosquito-borne illness	Symptom, today, health, born, mosquito, and effect	zika is a *mosquito borne* illness that does not present *symptoms* in many people. that is a very dangerous thing.
	#4 Abortion	Abortion, learn, worse, survive, guideline, and paper	zika virus, birth control and *abortion* our anti-woman laws will make this *worse*.
	#5 Fight the bite	Infect, fight, bite, affect, and death	only 1 in 4 people *infected* w/ #zika will show symptoms. *fight* the *bite*, destroy mosquito breeding sites #nobitenozika
	#6 Officials’ warning	Officials, control, disease, center, and researcher	health *officials* warn against exposure to zika at work the *centers for disease control* and prevention #atlanta
**Knowledge gap broader theme**		
	#7 Knowledge gap	People, expert, relate, Ebola, and cure	various *‘experts’* need to get up to speed on the zika+ front now. time is of an essence. many *people* are ‘behind the curve’.
**Zika characteristics broader theme**		
	#8 Symptoms	Fever, scarier, infect, eye, and first	zika symptoms– *fever*, rash, joint pain, and/or red *eyes*. most people *infected* typically don’t have symptoms though.
	#9 Dengue	Dengue, flu, rash, compare, cause, and malaria	*dengue* & zika have a *rash*, fever etc. 4 dengue strains increasing in ja. docs need to be careful #testedorsuspected
	#10 Fetal brain damage	Fetus, information, prevent, symptom, damage, and fetal	“why *fetal* tissue research is crucial to saving babies from zika new study uncovers ‘alarming’ *information* …”

In this case, the broader themes in Table 3 (public health messages, knowledge gaps, and Zika characteristics) highlight the neutral sentiments because the tweets in these themes were from public health experts and news agencies informing the public and thus are more likely to state facts than opinions. For example, the tweet “Officials: Zika-Infected Couples Should Postpone Pregnancy” is a statement from officials about postponing pregnancy during a Zika outbreak to help prevent babies born with birth defects. Some tweets were neutral even though they contained words with both positive and negative connotations because the sentiment of the tweet overall is neutral, such as this tweet “#voanews brazil scientists seek to unravel mystery of zika twins scientists struggling to unravel t….” Topics 1 through 6 all contained messages from public health agencies and were therefore labeled as public health messages. Topics 8 through 10 concerned characteristics of the Zika virus and thus were grouped together. Topic 7 did not belong in either category and was therefore made a separate theme. In summary, the neutral topics contained tweets from news agencies and public health officials. The negative sentiment topics also contained some tweets from news agencies and public health officials but additionally contained opinion tweets from the public.

### Topics from the Negative Sentiment

Before data analysis, we had chosen to focus on the topics from the negative sentiment category specifically in the symptoms category from our previous paper [4] as that was found to be critical for public health officials [31-35]. We chose to focus on negative sentiment tweets as this is what health officials will be most concerned with as there is greater need for intervention and information dissemination in these topics [31-35]. For example, a study by Glowacki et al [34] found that the Centers for Disease Control and Prevention (CDC) and the public expressed concerns about the spread of the Zika virus and that the CDC also focused on symptoms and education during a 1-hour live chat between the CDC and the public. Intense media focus on a topic, similar to the media focus during the Zika epidemic, causes concern among the general public [31]. Therefore, physicians and public health officials must address these concerns before they become entrenched in public discourse. The failure to act to the 2015 Ebola outbreak by the World Health Organization (WHO) and Centers for Disease Control (CDC) cost thousands of lives [32]. To prevent a similar failure, an intermediate-level response was needed to prevent overreaction while still taking adequate measures to respond to the Zika outbreak [32]. For example, during the Ebola outbreak, it was found that failure to engage communities had detrimental effects, whereas engaging communities helped curtail the outbreak [33]. The main ways to engage a community included involving family members in the care of loved ones in ways that did not put them at risk, tailoring global policies to local settings, using varied methods of communication, organizing regular meetings with the community, and identifying female and male community leaders to spread key messages. This is why public health officials in the CDC had the live chat with the public and posted information on social media as they gained new information concerning the Zika virus. The nature of the new symptoms associated with Zika could have encouraged fear and anxiety among the public [35]. Therefore, public health officials need to continue to disseminate preventative methods and information on how to address symptoms to help mitigate the panic. In addition, this was the category with the majority of the tweets (Figure 4). By understanding what is of concern to the public, officials can focus on targeting their messages to addressing these concerns. A methodology that seems to be effective based on our previous study [4] and the current LDA results is creating catchy phrases such as “Fight the Bite” or using phrases that elicit emotion such as the BBC article stating “Zika is scarier than initially thought.” Public health officials can focus on creating similar phrases to address all the topics of negative concern. The topic model results for negative sentiment are shown in Table 4. In the negative sentiment topics, there were 3 broader topics: *neural defects caused by Zika infection*, *abnormalities because of Zika infection*, and *reports and findings concerning the Zika virus*. Topics #1 *brain defects*, #2 *neurological effects*, #5 *fetal effects*, and #8 *Guillain-Barré syndrome* all concerned the nervous system. Topics #6 *Zika abnormalities* and #9 *Zika effects* were both related to abnormalities resulting from Zika infection. Topics #3 *initial reports*, #4 *Zika impact*, #7 *ultrasounds*, and #10 *dengue association* all concerned reports and findings concerning the Zika virus. There was significant overlap between topics #3 and #4 because they both addressed reports and findings concerning the Zika virus. However, topic #3 *initial reports* included tweets stating the locations where Zika is spreading, whereas topic #4 *Zika impact* included tweets concerning the BBC article that describes Zika as scarier than initially thought [3].

The broader themes in Table 4 (neural defects, Zika abnormalities, and reports and findings) were all negative because they addressed topics of concern for the general public. Before this outbreak, Zika was considered a mild illness with only 14 reported cases [2]. It was not until this most recent outbreak that Zika became associated with microcephaly, Guillain-Barré syndrome, and congenital Zika syndrome, all of which caused fear and concern across the globe [1,4,36,37].

Table 5 shows the percentage distribution of tweets belonging to each theme of the negative sentiment category. The tweets were evenly distributed across the topics, with the exception of topic #10 (dengue association). This is because people discussing this association are most likely epidemiologists and others in the public health field that understand antibodies, as seen in this tweet, “lab findings hint that #dengue antibodies intensify #zika infection=>leading to #microcephaly & gbs^a^? Evidence.”

**Table 4:** Negative sentiment topic modeling results grouped together based on the broader themes. The numbers reflect the relative size of the theme.

Topic		Words	Tweet
**Neural defects broader theme**		
	#1 Brain defects	Brain, microcephaly, baby, disorder, confirm, and cause	#zikavirus *confirmed* zika *causes brain* damage in *babies* born with *microcephaly* brain abnormalities in babies
	#2 Neurological effects	Severe, problem, immune, neural, death, and birth	human *neural* stem cells infected by #zika subsequently trigger an innate *immune* response that leads to cell *death*
	#5 Fetal effects	Brazil, fetus, shrink, development, disrupt, outbreak, and pregnancy	in #*brazil* zika eats away at *fetal* brain, *shrinks* or destroys lobes controlling thought & prevents *development*.
	#8 Guillain-Barré syndrome	Syndrome, rare, case, associate, cause, and microcephaly	*cases* of *rare* nervous disorder guillain-barre *syndrome* may increase if zika spreads via
**Zika abnormalities broader theme**		
	#6 Zika abnormalities	Brain, eye, abnormality, scientific, consensus, confirm, and relate	a9 zika associated complications for pregnancy include miscarriage, stillbirth, *brain abnormalities* and *eye* abnormalities. #reuterszika
	#9 Zika repercussion	Zikavirus, infect, child, adult, and fetal	researchers says that *zika virus infection* can stunt growth of *children*
**Reports and Findings broader theme**		
	#3 Initial reports	Report, puerto rico, infect, link, and defect	*puerto rico reports* first zika-*linked* birth *defect* ${3.1} *puerto rico reports* first zika-*linked* birth *defect*
	#4 Zika impact	impact, spread, reuters, mosquito, and scarier	#*reuters* zika *spread, impact 'scarier* than we initially thought' u.s. health official
	#7 Ultrasounds	ultrasound, doctor, baby, unborn, and infect	#chevycar *ultra sounds* missed zika *infection* until the one showing serious harm to her *baby*
	#10 Dengue association	Expert, warn, sound, dengue, causal, fetus, spread, and microcephaly	lab findings hint that #*dengue* antibodies intensify #zika infection=>leading to #*microcephaly* & gbs^a^? evidence

^a^GBS: Guillain-Barré syndrome.

**Table 5:** Percent distribution of tweets belonging to the ten themes in the negative sentiment category.

Theme	Distribution of tweets, %
Brain defects	12
Neurological effects	12
Initial reports	11
Zika impact	11
Fetal effects	11
Zika abnormalities	10
Ultrasounds	10
Guillain-Barré syndrome	9
Zika repercussion	9
Dengue association	5

## Discussion

In the discussion section, we will address one cause of tweets being misclassified with some examples. The 3 negative sentiment broader themes, *neural defects*, *Zika abnormalities*, and *reports and findings*, will then be explored and discussed in more detail.

### Classification Analysis

As seen in Table 2, classification is not 100% accurate, implying that some tweets were misclassified. We will focus on the negative tweets as those were the focus of our discussion. Some tweets were misclassified because of words such as *active*, *saliva*, *feds*, *busted*, *beast*, and *prenatal*, which were not seen by the model because the count of these words is less than the minimum count (set to 5) parameter given in the Word2vec model and hence were discarded. The minimum count was set to 5 (the default setting in Gensim) as words used fewer than 5 times do not add significant information to the analysis [38]. Adding more training data could improve these results; however, a study by Nakov et al annotated 6000 tweets and had similar F1 scores to our study [39]. As these words occurred fewer than 5 times, the algorithm was not able to identify these tweets as negative as it was not able to determine the words closer to these words. Examples of tweets that were incorrectly identified as negative are “#3tking Zika virus makes Rio Olympics a threat in #Brazil and abroad, #health expert says” and “#ap breaking cdc no longer any doubt that zika virus causes birth defects.” Examples of tweets incorrectly identified as positive are “#seattle major zika fail! feds busted for lazy response …” and “@DrFriedenCDC Scary how you could substitute prenatal alcohol in place of Zika!Same symptoms,hidden—YetCDC quiet.”

### Topic Model

In this section, we focus on the negative sentiment topics of neural defects, Zika abnormalities, ultrasounds, and dengue association. These themes and topics were chosen for discussion because they were topics of public concern, have been addressed by the CDC or WHO [36,40-44], and can be addressed by officials to help mitigate the concern. Zika impact was not addressed because it is the focus of our previous work [4]. Initial reports were not addressed as it is specific to this outbreak and officials and the public cannot wholly prevent the spread of the Zika virus.

### Neural Defects

Neural defects is a broader theme of concern for the public that needs to be addressed by public health officials to mitigate fear and concern because of the defects to the nervous system caused by Zika virus infection. For Table 4, topics #1 (*brain defects*), #2 (*neurological effects*), #5 (*fetal effects*), and #8 (*Guillain-Barré syndrome*) all concern the neural system. For example, topic #1, *brain defects*, points to brain damage in babies because of microcephaly as seen in this tweet “scans show extent of brain damage in babies with microcephaly associated with zika….” Microcephaly has been a topic of concern for the CDC as babies born with microcephaly will require assistance throughout their lifetime [40,45]. The topic *neurological effects* (#2) includes tweets discussing the death of neural stem cells, which leads to neurological disorders in humans [46], as seen in this tweet, “zika virus targets human cortical neural progenitors causing cell death & attenuated neural cell growth.” The topic *fetal effects* (#5) also addresses brain shrinking or brain damage but additionally the tweets discuss the destruction of the brain lobes that control thought, vision, and other functions in fetuses as seen in this tweet, “scans & autopsies show that zika eats away at the fetal brain. it shrinks or destroys lobes that control thought, vision & other functions.” *Guillain-Barré syndrome* (topic #8) is a sickness caused by damage to nerve cells. The tweet “human neural stem cells infected by #zika subsequently trigger an innate immune response that leads to cell death” includes information on how Zika can lead to damage of neural stem cells and causes a disease such as Guillain-Barré syndrome [47]. The reader can see how topics #1, #2, #5, and #8 all include information on neural issues following Zika infection but all focus on different issues and are, therefore, 3 separate topics. By looking at these tweets, public health officials can see the public is concerned about the neurological defects caused by Zika. Therefore, the next steps officials need to take is to focus on how to prevent mosquito bites, especially when pregnant, to prevent these neurological defects. The “Fight the Bite” campaign is an example of such an effort [44].

### Zika Abnormalities

Zika abnormalities is also an important broader theme to address because of the fear and concern of abnormalities and defects in infants because of Zika virus infection during pregnancy. In Table 4, the topics #6 (*Zika abnormalities*) and #9 (*Zika effects*) are both related to abnormalities because of Zika infection but include diverse problems. The topic *Zika abnormalities* (#6) describes various anomalies associated with the fetus and babies born with Zika infection as seen in this tweet, “birth defects linked to #zika now also incl hearing loss, vision problems, impaired growth, abnormalities in limbs.” These types of abnormalities are termed as congenital Zika syndrome by the CDC and includes a collapsed skull, eye scarring, severe muscle tension, and brain calcification [36,37]. The topic *Zika effects* (#9) focuses on the stunt in growth and development of children. Again, both of these topics concern abnormalities because of Zika infection but focus on 2 different abnormalities and are therefore kept as 2 distinct topics. By pushing prevention such as the “Fight the Bite” campaign, officials can help ease fears concerning these abnormalities.

### Ultrasounds

Ultrasounds is another important topic to address because initial ultrasounds fail to reveal microcephaly and other birth defects, leading to a false sense of security for a couple [41,42,48,49]. As previously stated, Zika is linked to microcephaly; however, ultrasounds were found to have high false-negative predictions regarding the presence of microcephaly during the first and second trimesters of a woman’s gestational period [48]. Therefore, the topic of *ultrasounds* is important to discuss because pregnant women may have a false sense of security after getting an ultrasound and Zika not being detected in their fetus in the early stages of pregnancy. The CDC states on their website that microcephaly is more readily detected late in the second trimester to early in the third trimester [41]. Researchers are also recommending that parents have a magnetic resonance imaging (MRI) procedure on their newborn’s head performed because some abnormalities are not apparent at birth but may be detected in an MRI [42]. To address the concern of detecting microcephaly before a baby is born, officials need to keep providing up-to-date information on ways to detect microcephaly and to keep striving to improve detection methods to help the public make informed decisions regarding their fetus.

### Dengue Association

Dengue association may explain why this Zika outbreak is associated with abnormalities and defects and previous infections were not, which is why it is an important topic to address [43,50-52]. Dengue is in the same family of viruses as Zika and is also spread by the same 2 mosquitoes as Zika [43]. If a person has been previously infected with 1 strain of dengue and then later gets infected with a different strain, they are at risk of developing severe dengue symptoms because of antibody-dependent enhancement (ADE) [50]. In the topic *dengue associations* (#10), scientists suspected and are starting to confirm that earlier illness of dengue enhances the chances of Zika infection also because of ADE [51,52]. The fact that this is in the negative sentiment category shows that the public is concerned with dengue interacting with Zika, which informs public health officials that their messages concerning this topic are being heard and causing adequate concern. Now that there is evidence that previous dengue infection enhances the chances of more severe Zika infection, public health officials need to proliferate this message across social media sites and encourage those with past dengue infection to continue to take precautions against mosquito bites.

### How to Address These Concerns

Now that public health officials know what the public is concerned about, they can focus on addressing these concerns. When an incident occurs, the normal tendency is to seek more information on the topic of interest [53]. This can be done by reading or listening to the news, performing internet searches, or communicating with others. Through this search for knowledge, concern can be diminished or enhanced, depending on the information gathered [54].

Complications related to processing can include the accuracy of the information shared, as at times the media is quick to report information without having all the facts or the reader may interpret the facts incorrectly [53]. Therefore, news agencies need to be more careful about what they publish and not use titles such as the BBC article did [3] that are meant to instill concern in the public. Deficiencies in communications among the media, the public, politicians, and scientists heightens concern [55]. For example, when nonexperts express views different from experts, public fear is heightened [56]. This is a difficult problem to address, as evidenced by the debate on vaccines and autism [57]. Experts need to keep putting factual information out there and also keep peer reviewing each other to make sure studies such as the one by Wakefield suggesting vaccines cause autism do not occur in the future [57]. Another common example is the level of information presented to the public. Scientists tend to use words the public does not understand, such as the word asymptomatic, causing a discrepancy between what is stated by public health officials and what the reader interprets. This can be addressed by scientists better explaining their work at an elementary school level.

The authors understand all of these suggestions are already being followed at some capacity by public health officials. However, there is always room for improvement.

### Limitations

The tweets in our analysis were limited to the English language, which limits the generalizability of the study. This is critical as South American countries were the first and hardest hit countries. Future studies can address this limitation by analyzing tweets in Portuguese and Spanish. The keywords used in data collection were Zika, Zika virus, Zika virus treatment, and Zika treatment. Therefore, tweets that refer to this disease in another language would be overlooked. Tweets that refer to the disease without mentioning it by name would also be overlooked.

Without prosody, contextual, and spectral cues, sarcasm is difficult to detect [58], all 3 of which are impossible to determine in a tweet. Some research has been done using lexical and pragmatic factors [59]; however, even the human annotators had less than 50% agreement on whether a tweet was sarcastic in this study. Clearly, if the ground truth is inconsistent, it cannot be modeled reliably with machine learning. The annotators in this study coded the tweets based on the sentiment they believed it expressed, with sarcasm being one of the causes of disagreement. However, very few tweets were considered to be possibly sarcastic in our dataset, thus limiting the effect.

Due to the short length of tweets and the large number of tweets collected, LDA has been previously shown to have some issues with overfitting, with the number of revealed topics exceeding the true number of topics [60]. We attempted to address both of these concerns in our study by combining positive tweets into a document, negative into another document, and neutral into a third document, thus making the datasets smaller and the topical domains more specific.

### Conclusions

Overall, the negative sentiment topics focused on neural defects and abnormalities caused by the Zika virus. As these tweets were categorized as negative sentiments, officials could see that the public was concerned with the symptoms caused by the Zika virus. As the public was concerned, officials could focus on spreading information encouraging prevention. Officials could also see that the top themes all concerned actual symptoms and defects and did not focus on misconceptions or misinformation that they needed to address. Moving forward, officials can also start informing the public that studies are providing evidence for the Zika-dengue interaction hypothesis. They should focus these messages in areas where dengue is endemic as they are the ones most at risk of the interaction causing more severe Zika infection.

When another Zika outbreak occurs, we predict similar concerns (such as microcephaly) about the neurological defects will be expressed on social media. Although our current framework would still be applicable, the unsupervised topics within the tweets would change. Specifically, the relevancy and sentiment classifiers (the supervised part of the system) would still be effective in detecting tweets specific to Zika and specific to the particular topic such as symptoms. However, when a preventative vaccine for Zika virus infection is created and/or new symptoms arise that are associated with Zika virus infection, the topics of concern would change depending on the current issue of concern at a particular time. As of August 2018, no licensed vaccines were available; however, several candidates are in various stages of development, and clinical trials have begun [61]. Once a licensed vaccine is available, we predict negative sentiment concerning Zika virus symptoms will decrease but most likely will not disappear. At that time, the methods utilized in this study will still be relevant, but the major topics in the negative sentiment category will likely change because of the decrease in concern, which would also be indicated by the increase in tweets in the positive or neutral categories.

On the contrary, if new symptoms for Zika were to develop or further complications for those born with neurological defects were discovered, the topics of concern in the negative sentiment category would change to reflect concerns specific to the new symptoms. During the most recent outbreak, scientists suspected that people who previously had a dengue infection experienced worse symptoms from Zika than those who had not been previously infected with dengue [62]. Previous infection with a similar virus to Zika, such as West Nile, may cause new symptoms like we saw with dengue and Zika [51,52].

Our study is also useful for those that want to perform sentiment analysis with an epidemic, pandemic, or bioterrorism attack. Sentiment analysis is complex as most sentiment analysis tools just use the individual word polarities for measuring sentiment and generate an automated scoring mechanism based on these polarities to rate the sentiment levels of each tweet. This fails to incorporate the contextual information that needs to be incorporated for topic-specific sentiment analysis in this domain [5]. Scientific topics especially require manual labeling as science words with negative sentiment can actually have a positive context as seen in this tweet, “Obama diverts Ebola funds to fight Zika; Florida leads nation in case…” The word *fight* would typically have a negative connotation but has a positive one in this tweet. Some examples of other words that are typically considered negative but are actually positive when discussed under the context of epidemics are *combat*, *prevent*, and *impair*. If tweets containing these words were categorized using a sentiment word bank, they would have been incorrectly categorized as negative. This is an important issue because it does not correctly represent the public’s feelings and may cause experts to believe the public is not as concerned about Zika symptoms if some of the negative tweets were misclassified as positive/neutral. Therefore, we used a manual labeling process where an entire tweet was assigned to a sentiment category by 2 domain experts. We believe that this need for a combination of data science and domain expertise is what makes our study challenging and interesting.

This is one of the first studies to address Zika sentiment classification using Twitter. Using such a system allows public health officials to ascertain public sentiment concerning disease outbreaks and address concerns in real time.

### Future Work

Future studies could analyze the change in sentiment over time to see if the number of negative tweets decreases as the outbreak subsides and more advances in treatments are discovered. Studies could also look at sentiment by gender or geographic location. Both are prudent because of Zika’s effect on fetuses and its comparative prevalence in equatorial regions, respectively. We would also suggest future studies to leverage other sources of information, such as other social media sites, newspapers, and blogs. Similar methodologies could also be applied generally to future pandemics and epidemics to ascertain public sentiment.

## Abbreviations

ADE: antibody-dependent enhancement
ASCII: American Standard Code for Information Interchange
CBOW: continuous bag of words
CDC: Centers for Disease Control and Prevention
LDA: latent Dirichlet allocation
MRI: magnetic resonance imaging
RQ: research question
WHO: World Health Organization
3D: 3-dimensional
